# 

**Published:** 1898-09-17

**Authors:** 


**Notices of Births, Deaths, and Marriages are inserted in this column at a charge of 2s. 6d. for an announcement not exceeding four lines.**

### NOTICE TO CORRESPONDENTS.

All MS., Letters, Books for Review, and other matters intended for the Editor should be addressed The Editor, "The Hospital," The Hospital Building, 28 & 29, Southampton Street, Strand, W.C.

The Editor cannot undertake to return rejected MS., even when accompanied by stamped directed envelope.

**Notice to Advertisers and Subscribers.**

All Advertisements, Orders for Copies of The Hospital, and Business Communications should be addressed to **THE MANAGER, (Not to The Editor),** The Hospital, The Hospital Building, 28 & 29, Southampton Street, Strand, London, W.C.

The Cost of Subscription, post free, is as follows:—Per week, 2½d. per quarter, 2s. 8d.; per half-year, 5s. 3d.; per annum, 10s. 6d. Foreign:—Per annum, 15s. 2d.; payable, in all cases, in advance.

**NOTICE.—Vol. XXIV. of "The Hospital" (April, 1898, to September, 1898), in handsome cloth binding, will shortly be on sale at the Office of this Journal, price 7s. 6d. Cases for binding the Numbers contained in this Volume, 1s. 6d. Index to Vol. XXIV., 2½d., post free.**

**The Monthly Part for October will be ready on October 1. Price 6d., by post 8d.**

### Scraps and Gleanings.

Abingdon Hospital Sunday will be held this month.

The Leatherhead Sunday Church Parade realized a sum of £19 8s. this year.

The Seamen's Hospital Society are legatees of £500 under the will of the late Mrs. M. A. Perrens Perrens.

The Salford Royal Hospital has received upwards of £650 this year from the Salford Hospital Sunday and Saturday Funds.

Over £60 was collected in Ilfracombe in aid of the Tyrrell Cottage Hospital through the annual street collection, concert, &c.

Sir Ralph Thomson will preside at the annual dinner of past and present students of Middlesex Hospital, to be held on October 3rd.

During the past quarter 1,060 medical and surgical patients have been treated in Middlesex Hospital, and 10,708 in the out-patient department.

The work of erecting the new ward at the isolation hospital, Theydon Garnon, near Epping, will shortly be commenced. Tenders are now being asked for.

The Ross Cottage Hospital has benefited to the extent of £100 under the will of the late Mr. James Llewelly, of Ross, who also bequeathed £50 to the dispensary.

At Munich, in Germany, there is a hospital which is supported by the sale of old steel pen-nibs, collected from all parts of the country, which are made into razors, knives, &c.

The plans for the Victoria Convalescent Home for Surrey Women at Bognor have been approved by the Bognor Council, and a start will be made with the work very shortly.

The work in connection with the erection of the nurses' home of the Consumption Hospital, Brompton, will be proceeded with as soon as possible, the plans having been now decided upon.

The Worshipful Company of Leathersellers recently made a grant of £10 10s. to the funds of the After Care Association for Poor Persons Discharged Recovered from Asylums for the Insane.

The Harrison Almshouses, Matlock, are now ready for occupation. They are the gift of Miss Harrison, who has given in all about £8,000 to Matlock for the buildings, which are perpetually endowed.

Madame Calve, the great prima donna, has founded on her estate, near Millau, in the department of the Aveyron, a sanatorium for poor children, which is to be placed in charge of the nuns of the local convent.

The Aberdeen Royal Infirmary has received £600, and the convalescent hospital in connection with it has had £100 from the Friendly Societies' Hospital Saturday Committee this year—a very handsome donation.

The Managing Committee of the Llanelly Hospital have decided to reduce the number of beds from 31 to 16, this step being forced upon them by reason of the unsatisfactory state of the finances of the institution.

The Porth Cottage Hospital, which is kept up by the workmen, has for the present been closed for want of funds. This circumstance is stated to be due in large measure to the postponement of the Porth annual Eisteddfod.

A plan which might well be followed by the authorities of other popular seaside resorts, is that which is pursued by Eastbourne, of having band performances of sacred music, when collections are made on behalf of a local hospital.

During the months from August, 1897, to August, 1898, 107 patients were received into the Royal Alexandra Children's Hospital and Convalescent Home at Rhyl, from Birmingham and the immediate neighbourhood, but there were only 13 subscribers from Birmingham to the hospital.

Those who have watched the doings of the Bedford Centre of the St. John Ambulance Association, are pleased at the recognition of her energetic devotion on its behalf which has been conveyed to Mrs. Paine by the Secretary of the Order of St. John of Jerusalem, who has intimated that Mrs. Paine has been enrolled an Honorary Associate of the Order.

Considerable enthusiasm was displayed in connection with the recent Friendly Societies' Parade at Brighton in aid of the local medical charities. There were trolleys bearing a kind of *tableau vivant*, one depicting two ladies as nurses attending a small patient, and another representing "The Bride." There was a very ingeniously decorated cycle representing a brigands' cave, and there were banners and bands galore.

Owing in a great measure to the expense of maintaining the enlarged Aberdeen Infirmary and the increase in the number of patients under treatment, the expenditure for the first seven months of this year has exceeded the income by £1,300. The public are urgently requested to increase their subscriptions in proportion with the increased amount of work which has to be done owing to the larger number of patients now than formerly, there being a daily average increase of upwards of 50. Despite this the ordinary income for the first seven months of 1898 is less than what has been received during the same period for the last five years.

The Barnsley Hospital Saturday and Sunday Committee are ambitious, and they deserve the success which crowns their efforts. At the open-air musical festival held on Sunday, August 21st, in aid of the Beckett Hospital and Dispensary, the orchestra and performers numbered about 300, the programme including selections from "Samson" and Mozart's "Twelfth Mass." We hope that the pecuniary result of this effort will be as satisfactory as on the last occasion. Last year the Barnsley Hospital Saturday and Sunday Committee raised the splendid sum of £120, which was all handed over to the hospital with the exception of £120—a very small percentage for expenses.

### The Student of Medicine.

### APPOINTMENTS.

B. M. Bone, M.B., C.M.Edin., appointed Medical Officer of Health to the Towyn Urban District Council. W. J. Bowden, M.B., Ch.B.Vict., appointed Medical Officer for the Glossop District of the Glossop Union. F. Brannan, M.B., B.Ch., &c., R.U.I., appointed Medical Officer, Castledermot Dispensary, co. Kildare. H. Connop, L.R.C.P., L.R.C.S.Edin., appointed Medical Officer for the Brailsford District of the Ashbourne Union. C. E. Durrant, L.R.C.P.Lond., M.R.C.S.Eng., appointed Resident Medical Officer to the National Sanatorium for Consumption and Diseases of the Chest, Bournemouth. C. Hairison, M.D., appointed Medical Officer of Health to the Bracebridge Urban District Council. A. S. Hedley, M.B. and B.S.Durh., appointed Medical Officer for the (East) Rothbury District, and Medical Officer to the Rothbury Union Workhouse. J. M. Hughes, M.B., C.M.Edin., appointed Medical Officer for the Workhouse and the Ruthin District of the Ruthin Union. D. K. Muir, L.R.C.P., L.R.C.S.Edin., appointed Medical Officer and Public Vaccinator for the Walker District of the Tynemouth Union. A. H. Pirie, B.Sc.Edin., M.B., Ch.B., appointed Resident Medical Assistant to the Dundee Royal Infirmary. G. H. Ruston Hairison, M.B. and C.M Edin., appointed Senior Resident Surgeon to the Nottingham General Dispensary. G. Sharp, M.D.Edin., appointed Honorary Medical Officer to the Beckett Home, Meanwood, Leeds. J. B. Strang, M.B., appointed Medical Officer for the Humshaugh District of the Hexham Union. H. J. Taylor, M.R.C.S.Eng., L.R.C.P.Lond., appointed Junior House Surgeon to the Manchester Royal Eye Hospital. Dr. Thomas, appointed Medical Officer for the Guilsfield District of the Llanfyllin Union. G. F. Whyte, M.B., C.M.Edin., appointed Resident Medical Assistant to Dundee Royal Infirmary. J. R. R. Pollock, M.R.C.S., L.R.C.P.Lond., appointed Medical Officer of Health to the Tiverton Rural District Council. H. A. Powell, M.B.Adel., appointed Medical Officer at Kadina, South Australia. Mr. R. A. Smith, appointed Medical Officer for the Finchingfield District of the Braintree Union. A. E. Street, L.R.C.P.Lond, M.R.C.S., appointed Resident Medical Officer of the Dyffryn Aled Branch of the Cheadle Royal Lunatic Hospital. J. Teare, M.B.Vic.Univ., appointed Honorary Physician to the Wellington Hospital, New Zealand. Julia Thomas, M.B.Syd., appointed Resident Surgeon to the Children's Hospital, Sydney. A. H. Tubby, M.S., M.B., appointed Surgeon to In-patients on the Honorary Medical Staff of the Evelina Hospital for Sick Children. W. R. Walton, M.R.C.S., L.R.C.P.Lond., appointed Medical Officer for the Melton District of the Wolstanton and Burslem Union. Dr T. Wilson, appointed Health Officer at Cuddingwarra, Western Australia.

